# The role of self-efficacy beliefs in dealing with misinformation among adolescents

**DOI:** 10.3389/fpsyg.2023.1155280

**Published:** 2023-05-18

**Authors:** Marinella Paciello, Giuseppe Corbelli, Francesca D’Errico

**Affiliations:** ^1^Faculty of Psychology, Uninettuno University, Rome, Italy; ^2^Department of Education, Psychology and Communication Studies, University of Bari “Aldo Moro”, Bari, Italy

**Keywords:** adolescence, misleading news, online sharing, self-efficacy, self-regulation

## Abstract

The present study aims to understand the processes involved in misinformation among adolescents by examining the role of self-efficacy beliefs in dealing with misleading news. Specifically, we argue that the perceived capability to analyze and reflect critically on the reliability of online information sources should be stayed with the perceived self-regulatory capability to resist online social pressures to share unverifiable news. Moreover, we posited that specific online self-efficacies beliefs can be promoted by the capabilities related to regulating emotions and reflecting on new problems. In a sample of 273, we tested a path analysis model. The results attest that self-efficacy beliefs in dealing with online misinformation refer to specific capabilities: an active one, related to checking the sources of the news in order to validate their content, and an inhibitory one, related to the capability to refrain from sharing the news that seems unreliable. Moreover, self-efficacy beliefs in self-control during online interaction spreading misleading news are supported by cognitive reflective capability and self-efficacy in regulating negative emotion. The relationship between active self-efficacy related to fact-checking and sharing misleading news is not significant. The role of regulation in sharing misinformation during activated online dynamics is discussed.

## 1. Introduction

The literature on fake news among adolescents has highlighted the youth’s exposure and vulnerability in dealing with fake and misleading news as well as their propensity to easily share them (e.g., Herrero-Diz et al., 2020). It seems that, although youth are aware of the lack of credibility of information on social networks, they give little importance to verification strategies such as the control of authors or the sources of the news (Papapicco et al., 2022). Moreover, if fake news attracts their attention, they impulsively share it even knowing that the information could be not reliable (Herrero-Diz et al., 2021). The sharing of fake news can be driven by adolescents’ need to feel included in the social group or/and to inform others of their interests (Beyens et al., 2016; Notley and Dezuanni, 2019). However, empirical studies in this area are rare and the majority have been conducted on adulthood samples (Pennycook and Rand, 2021). Moreover, the possible processes involved in sharing fake news have yet to be clarified, especially in the new generation of teenagers born after the advent of social networks and massively active in their use.

The present study aims to understand the processes involved in misinformation, i.e., the unintentional diffusion of incorrect or false information, but transmitted with the conviction of its truth (Ireton and Posetti, 2018), with particular attention to misleading news that relies on the hyperpartisan description of the event in the adolescents. In understanding the sharing of misleading news, we examine the role of self-regulatory self-efficacy beliefs in dealing with misinformation, especially during online peer interactions. Self-efficacy represents a central mechanism in the exercise of human agency expressing the individual judgment on personal self-regulatory capabilities needed to exercise control over one’s own thought processes, motivation, and action (Bandura, 1992). A large body of longitudinal studies has proven the protective power of self-efficacies beliefs related to different domains of functioning in promoting adaptive behavior (Bandura et al., 1996; Caprara et al., 2006) and counteracting negative ones during adolescence (Bandura et al., 1999, 2001).

Following this literature and in accordance with Social Cognitive Theory (Bandura, 1992) we argue that also in the case of misinformation self-efficacy should play a crucial function in dealing with misleading news since it can capture the strength of those specific capabilities required to regulate online behavior under certain online circumstances.

*H1*: regulatory self-efficacy in sharing misinformation is negatively linked to the online sharing of misleading news.

To date, the existing measures of self-efficacy developed in understanding online sharing behavior concern “constructive” forms of online behavior that are those self efficacies beliefs related to sharing competencies and knowledge at work in a collaborative way by experts (Chiu et al., 2006; Hsu et al., 2007; Zhang et al., 2017). In some cases, specific self-efficacy constructs related to the individual’s belief in her ability to evaluate and identify digital misinformation were theorized (Hocevar et al., 2014; Khan and Idris, 2019). These findings, which involve only samples of adults, suggest that self-efficacy related to fact-checking skills may not necessarily be sufficient to counteract the sharing of misinformation—especially in inexperienced users. In the case of adolescents, we argue that in order to examine the role of a specific form of online self-efficacy in hindering information sharing, it is certainly important to consider their abilities to monitor, search, analyze and reflect critically on the reliability of the online information sources. Most educational programs stressed the opportunity to teach these digital media competencies (McGrew et al., 2019). However, we also believe that this active ability to navigate online information must be accompanied by an inhibitory capability to refrain from sharing unverifiable news under online sensitive social circumstances. More specifically, we hypothesize that online sharing could be induced also by peer online dynamics or/and online social and interpersonal incentives that are extremely important during adolescence (Steinberg and Monahan, 2007). Thus, as in the case of “offline” misbehavior such as skipping school, drinking alcoholic beverages, or smoking (Bandura et al., 2001), adolescents can benefit from their perceived capability to self-control and resist online social pressures to impulsively share unverifiable news.

In addition, according to social cognitive literature (Bandura et al., 2003) we posit that these task-specific online self-efficacies beliefs can be promoted by more general perceived capabilities related to regulating emotions.

*H2*: regulatory emotional self-efficacy is positively associated with regulatory self-efficacy in sharing misinformation.

If adolescents have higher levels of perceived emotional regulation skills, they should be more likely to engage in behaviors in line with their specific goals in relation to misinformation rather than being influenced by emotional reactions triggered by peer pressure or the news content itself (Bandura, 1977). We further hypothesize that the individual propensity to reflect analytically on information (rather than choosing an impulsive response) should have a positive influence on the misinformation-specific self-efficacies beliefs.

*H3*: as the propensity to engage in analytical reasoning increases, regulatory self-efficacy in sharing misinformation also increases.

**Figure 1:**
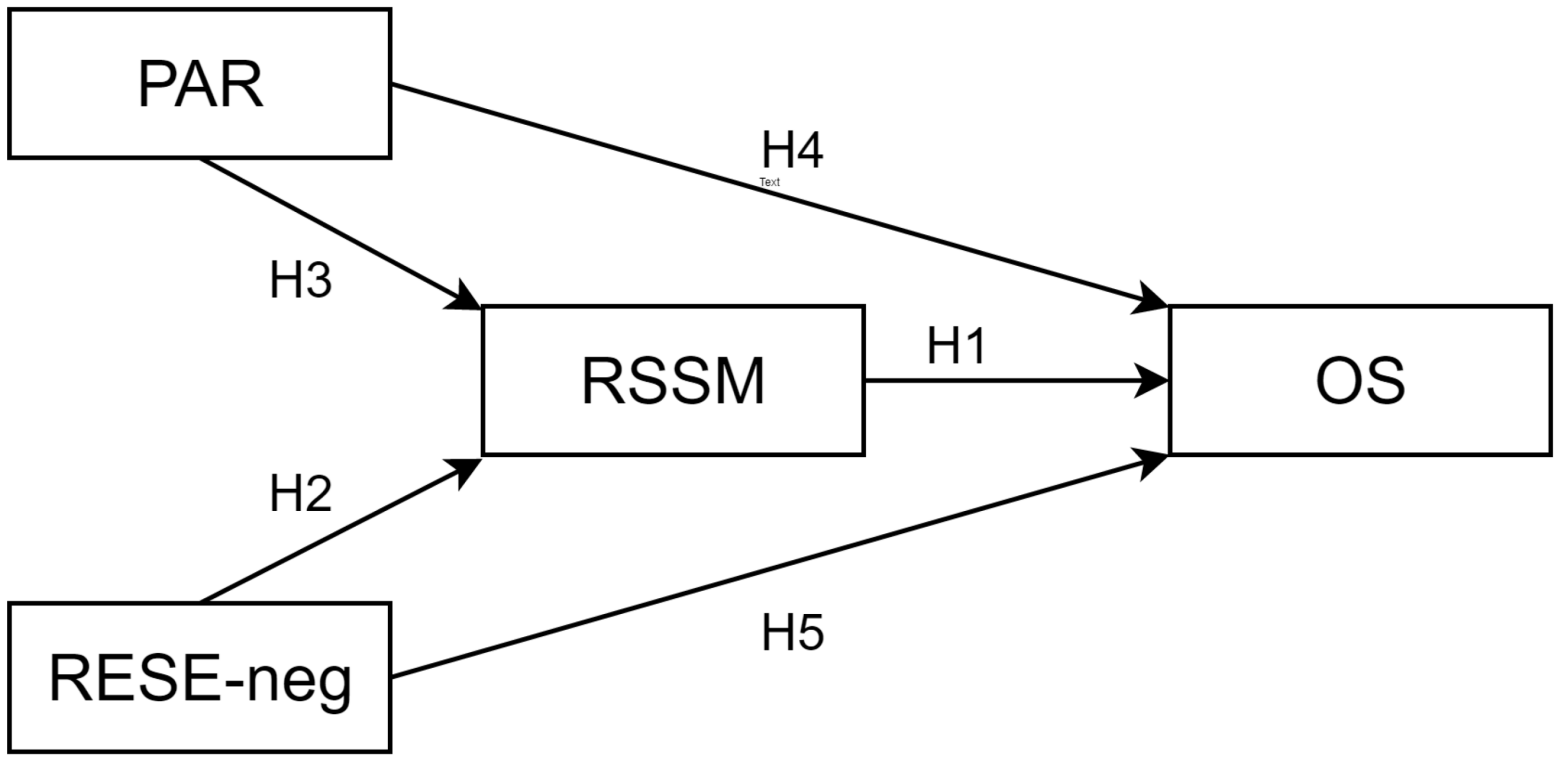
Hypothesized theoretical model. RESE-neg, Regulatory Emotional Self-Efficacy (negative emotions); PAR, Propensity to engage in Analytical Reasoning; RSSM, Regulatory Self-Efficacy in Sharing Misinformation; OS, Online Sharing.

Given that the protective importance of this “effortful processing” against misinformation is already acknowledged (Pennycook and Rand, 2021), this basic ability could also be linked directly to online sharing; similarly, given that the online sharing of misleading news occurs in emotionally activating circumstances (Preston et al., 2021), we hypothesize also a positive association between regulatory emotional self-efficacy and online sharing (see Figure 1).

*H4*: as the propensity to engage in analytical reasoning increases, online sharing decreases.

*H5*: regulatory emotional self-efficacy is negatively linked with online sharing.

Overall, the present study can contribute to the literature by highlighting the role of self-efficacy, a well-recognized adaptive dimension—related to the development of cognitive, emotional, and behavioral regulation—still overlooked in this area of research. Moreover, the focus on this agentic dimension can be crucial in designing educative and preventive programs aimed to develop basic and specific personal resources to counteract online dysregulated communications.

## 2. Method

### 2.1. Participants

The current study is part of an ongoing European project involving several countries aimed at investigating the phenomenon of misinformation on social networks in adolescence; therefore, students in the first 2 years of a major high school in the metropolitan area of Rome were chosen as a convenience sample. In total, 273 students participated in the research of which 224 were male (82%) while their ages ranged from 13 to 17 (*M* = 14.5, SD = 0.7). Regarding daily time spent on social networks, the majority of respondents (34.6%) reported a habitual interaction with a social network for more than 3 h each day, with the female fraction of the sample significantly engaging in activities on social networks more frequently than their male counterparts [*t*(261) = 2.0, *p* = 0.048].

### 2.2. Procedure

The battery was structured through the scientific markup language Inquisit (2021) and administered through the same psychological measurement software. The research project was fully endorsed by the Headmaster and the Board of Teachers, and all the teachers involved in the project received an information sheet detailing the research procedure and the measurements included in the questionnaire. All the procedures followed the Helsinki ethical principles and ethical codes of AIP (Italian Psychology Association), and the research project was approved by the Ethics Committee of the University to which one of the authors is affiliated with the reference code ET-22-01. All participants’ families were required to return a signed copy of the informed consent form by which students and their caregivers were informed in detail about the objectives of the study, its methodology, and the procedure for processing personal data in accordance with current legislation.

### 2.3. Measures

#### 2.3.1. Regulatory emotional self-efficacy

The scale of Regulatory Emotional Self-Efficacy (RESE-neg), designed by Caprara et al. (2008) was used to assess a person’s perceived ability to functionally manage their negative emotional states (i.e., despondency-distress, anger-irritation) to achieve an intended goal without being overwhelmed by them. The negative subscale consists of 8 items. Each item is rated on a 5-point Likert scale ranging from 1 (“Not at all”) to 5 (“Completely”) measuring the perceived ability to manage a different aspect of one’s own negative emotions. Cronbach’s *α* was 0.81.

#### 2.3.2. Propensity to engage in analytical reasoning (cognitive reflection test-2)

The Cognitive Reflection Test was used to measure the individual propensity to analytically ponder an issue, taking the time to further elaborate on the problem instead of impulsively giving an intuitive (but incorrect) answer. The CRT-2 revised version was preferred (Thomson and Oppenheimer, 2016), because of the lower numeracy skills required to provide the correct answer (Böckenholt, 2012; Sinayev and Peters, 2015). Cronbach’s α for the coded open responses was 0.6.

#### 2.3.3. Regulatory self-efficacy in sharing misinformation

Regulatory Self-Efficacy in Sharing Misinformation (RSSM) was assessed by 8 items specifically created for the present study. The scale aims at assessing the perceived ability of youngsters to cope with online disinformation, taking into consideration an inhibitory and an active factor in line with social cognitive literature (Bandura, 1991). The inhibitory factor (5 items) should assess the perceived ability of adolescents to refrain from sharing a piece of news even when it would benefit them or they feel the urge to do so, while the active factor (3 items) is aimed at measuring the perceived ability of teenagers to take action themselves by inquiring and investigating the veracity of a piece of news. Participants were required to rate their level of perceived capability (“When facing a piece of news that seems dubious to you, how capable do you think you are of…”) by rating it on a 5-point Likert scale ranging from 1 (“Not at all”) to 5 (“Completely”). Reliability and factorial structure for this scale are presented in the result section.

#### 2.3.4. Online sharing of misleading news

Instead of investigating the behavioral intention to share a news item through a decontextualized question, a specific behavioral variable to this aim was created in a simulative social network context. First of all, misleading news articles were designed according to the findings of a series of focus groups with teenagers of an age range equivalent to that of the target population, as well as with the help of professional journalists. In particular, we decided to take into account misleading news related to moral violations toward individuals or groups (e.g., real or symbolic aggressions). The misleading news included exaggerations (e.g., superlatives), sensationalist language, arbitrary negative evaluations of the responsible, and one-sided descriptions of the fact (Litovsky, 2021).

Then, the fake news stories were implemented in the form of realistic Instagram screenshots specifically designed to look like they were captured by one of the most commonly used smartphones among teenagers (see Supplementary material), thus emulating one of the most common ways among teens to exchange information, news, contextualized images, notifications from social networks, or comments to specific posts (Pennycook and Rand, 2021). In this ecologically relevant context for adolescents, the behavioral intention to share the same news item was subsequently detected on a scale ranging from 1 to 5.

### 2.4. Planned analyses

In the first place, an Exploratory Factor Analysis was used to assess the factorial structure of the newly developed RSSM scale. Secondly, in order to test the proposed relationships between the variables of interest, a path analysis was performed. Analyses were conducted in R v.4.2.0 (R Core Team, 2021) mainly with the *psych* package v.2.2.5 (Revelle, 2022); in addition, the *lavaan* package v.0.6-7 (Rosseel, 2012) was employed for path analysis.

## 3. Results

### 3.1. Regulatory self-efficacy in sharing misinformation scale

Considering the distribution of individual items of the RSSM scale, both skewness and kurtosis were always below the ∣1∣ cut-off; internal consistency was assessed, returning a Cronbach’s alpha of 0.81 for the whole scale. Kaiser-Meyer-Olkin’s measure of sampling adequacy was 0.86, and Bartlett’s test of sphericity was significant [*χ*^2^ (28) = 546.10, *p* < 0.001]. Given these overall indicators, it was deemed appropriate to proceed with the Exploratory Factor Analysis. Parallel analysis (using both PCA and PAF) suggested a two-factor solution; exploratory factor analysis, therefore, showed that all loadings associated with the two factors were above the cut-off of 0.30 (see Table 1; extraction method: principal axis factoring; rotation method: oblimin). The factor correlation matrix indicated a moderately strong correlation (*r* = 0.65) between the two factors, with the first factor accounting for 27% of the total variance in the observed variables and the second factor accounting for 14% of the total variance. Together, the two factors accounted for 41% of the total variance in the observed variables.

**Table 1:** Factor loadings and communalities for oblimin rotated two-factor solution for the 8 RSSM items.

	Factor loading		
	1	2	Communality
1. Avoid spreading it even if you risk being excluded from the group	**0.57**	0.19	0.50
2. Not spread it even when you could gain so much visibility by doing so	**0.51**	0.21	0.45
3. Refrain from spreading it even when everyone else is doing it	**0.74**	0.00	0.55
4. Tell others you have doubts about the news even when no one else does	0.25	**0.36**	0.31
5. Provide verified sources even when no one wants to do so	0.00	**0.69**	0.48
6. Refrain from sharing it even when it is in line with what you think	**0.52**	0.00	0.28
7. Refrain from sharing it even when you have a strong doubt that it is false	**0.70**	−0.12	0.39
8. Investigate sources even when you do not feel like it	0.06	**0.49**	0.29

Factor loadings above 0.30 are in bold.

### 3.2. Path analysis

Summary statistics and zero-order correlations for the variables of interest are shown in Table 2. Although skewness and kurtosis for all the relevant variables were smaller than the ±2 cut-off (Trochim and Donnelly, 2006), Mardia’s test for multivariate normality (Mardia, 1970) resulted in a statistically significant departure from multivariate normality for skewness (90.6, *p* < 0.001). Therefore, path analysis was performed with maximum likelihood estimation with robust (Huber-White) standard errors.

**Table 2:** Descriptive statistics and zero-order correlations for the relevant variables.

	*M*	SD	1	2	3	4
1. RESE-neg	3.16	0.84	–			
2. PAR	1.59	0.30	−0.005	–		
3. RSSM-inhib	3.51	0.90	0.223***	0.134*	–	
4. RSSM-act	3.40	0.91	0.127*	0.102	0.547***	–
5. Online sharing	2.04	1.25	−0.052	−0.111	−0.154*	−0.056

RESE-neg, Regulatory Emotional Self-Efficacy (negative emotions); PAR, Propensity to engage in Analytical Reasoning; RSSM-inhib, Regulatory Self-Efficacy in Sharing Misinformation (inhibitory factor); RSSM-act, Regulatory Self-Efficacy in Sharing Misinformation (active factor).

*p < 0.05, ***p < 0.001.

**Figure 2:**
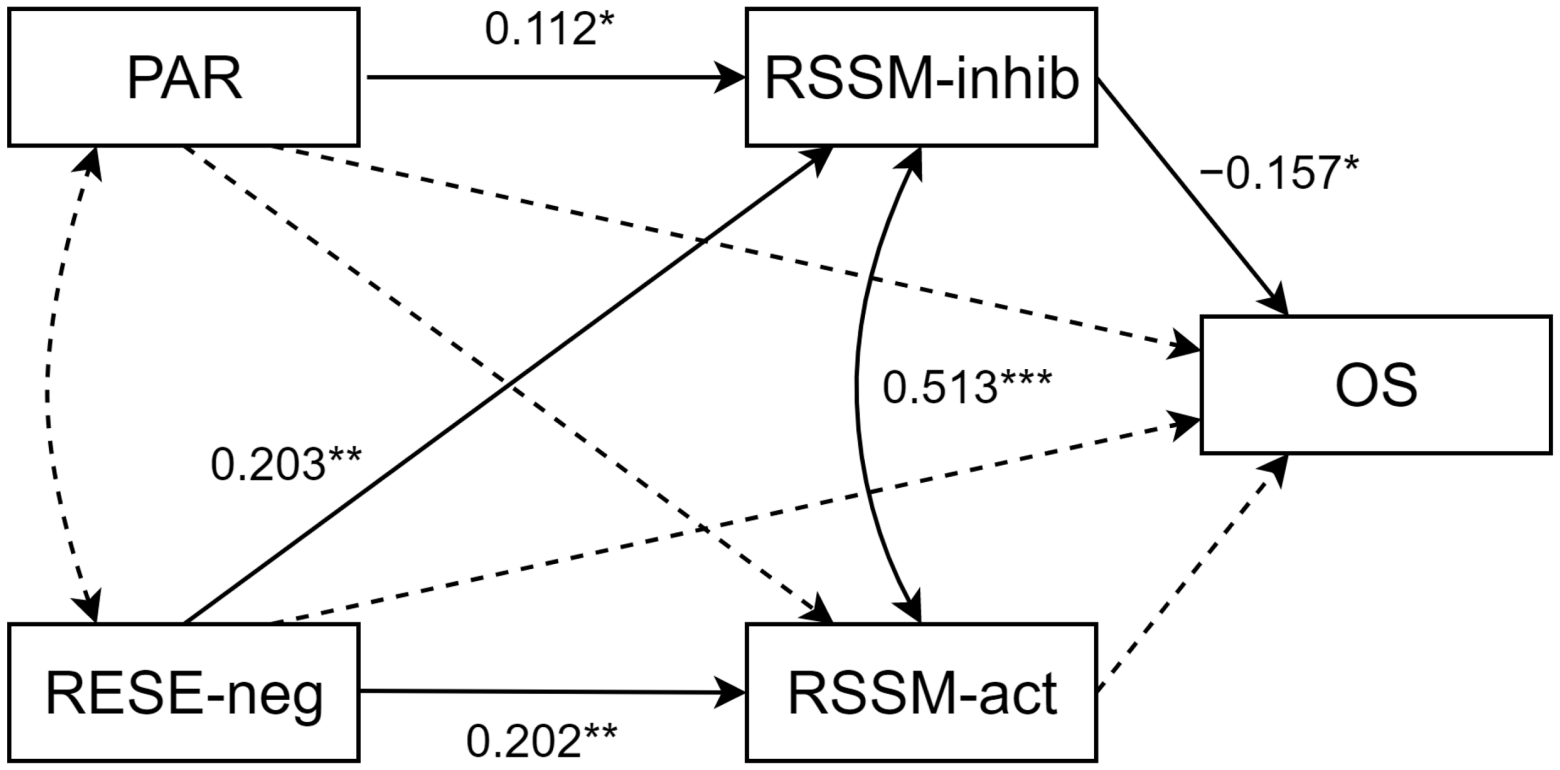
Path diagram: Robust maximum-likelihood parameter estimates for hypothesized model. The displayed estimates are the standardized coefficients. The effects of controls (gender and daily time on social networks) are not shown for the sake of clarity. Solid lines indicate paths significant at *p* < 0.05; dashed lines indicate non-significant paths. RESE-neg, Regulatory Emotional Self-Efficacy (negative emotions); PAR, Propensity to engage in Analytical Reasoning; RSSM-inhib, Regulatory Self-Efficacy in Sharing Misinformation (inhibitory factor); RSSM-act, Regulatory Self-Efficacy in Sharing Misinformation (active factor). *p < 0.05, **p < 0.01, ***p < 0.001.

The results of the path analysis are presented in Figure 2; for the sake of parsimony, the effects of controls (gender and daily time on social networks) are not shown. According to Kline’s cut-off values (2015), the proposed model is found to be plausible on the basis of the fit indices [*χ*^2^ = 11.414, df = 9, *p* = 0.248; CFI = 0.98; TLI = 0.96; RMSEA = 0.03 (90% CI = 0.000–0.083), *p* = 0.668; SRMR = 0.044].

Only the inhibitory RSSM factor is negatively linked to the behavioral intention to share the piece of misleading news online (H1), while emotional self-efficacy accounts for variance in both inhibitory and active RSSM (H2). Reflexive ability is positively linked only with the inhibitory factor of RSSM (H3). No significant effects from the two exogenous variables to online sharing were found (H4, H5). As for the control variables, the effect of gender significantly impacts the individual belief toward the perceived regulation of negative emotions (*β* = −0.255, *p* < 0.001) and the active aspect of the RSSM (*β* = 0.127, *p* = 0.015). Also, the number of hours spent on social media is negatively linked to the inhibitory aspect of the RSSM (*β* = −0.128, *p* = 0.015).

## 4. Discussion

The present findings show that self-efficacy beliefs in dealing with online misinformation can hinder misleading news sharing among adolescents. In particular, this self-efficacy refers to distinct perceived capabilities: (1) the proactive one related to checking the online sources of the news in order to validate their content; (2) the inhibitory one instead related to the capability to refrain to share a piece of news that seems unreliable, even when this could produce some benefit for adolescents. However, the former is not significantly related to online misleading news, supporting the previous findings on the young adulthood samples (Khan and Idris, 2019); differently, the latter can play a protective role in contrasting the spread of misinformation among adolescents. These results suggest that for adolescents, the perceived fact-checking ability may probably not be sufficient to deal with misinformation, as in the case of non-expert users (Zhang et al., 2017). However, since this is not always the case (Hocevar et al., 2014), future studies should examine how adolescents build these beliefs related to the evaluation of online information and if their perceptions are effectively rooted in efficacious experiences. In the case of the capability of resisting sharing fake news, it seems that adolescents’ behavioral regulation in an online interpersonal setting can make a difference. The role of self-regulatory self-efficacy in contrasting misbehavior, in general, has already been demonstrated (Bandura et al., 2001). As in the case of offline misbehavior, the capacity to refrain from acting online behavior that is recognized as improper but socially tempting is important also in the online context. Moreover, in an unexpected way, both cognitive reflection and self-efficacy in regulating negative emotion show no significant relationship with fake news sharing—despite their general influence on active and inhibitory self-efficacy beliefs. Arguably, as is the case with negative online behaviors at this stage of life (e.g., cyberbullying, Paciello et al., 2020), contextualized self-regulatory processes related to online dynamics may be independent of personal vulnerability, but influenced by the affordances of social media.

In terms of practical implications, technologies can support self-efficacy beliefs, which are malleable and influence self-determined behavioral change through direct or mediated experience (as in this case); indeed, technology has been shown to have the potential to enhance regulation and critical thinking skills (Kitsantas et al., 2019). In the case of adolescents, social networks are the new social laboratory within which they can learn behavioral regulation strategies (modeling) even from “positive” peers who provide alternative models of coping with online misinformation, but they can also have mediated experiences through games or targeted applications (D’Errico et al., 2023) that challenge and develop their ability to regulate themselves before sharing unreliable news.

Finally, although we used an ecological simulation approach to measure online sharing intention, the data were from a single source; this is a potential limitation regarding the reliability of the reports. Another limitation of the study is related to the fact that we did not include other important factors associated with misinformation in the model, and that we conducted the research on a local sample of adolescents. These limitations could be addressed by replicating the present study in different cultures with larger adolescent samples by integrating other important factors moderating the relationship between self-regulatory capabilities and online misinformation within a longitudinal framework.

## Data availability statement

The raw data supporting the conclusions of this article will be made available by the authors, without undue reservation.

## Ethics statement

The studies involving human participants were reviewed and approved by Ethics Committee of the University of Bari “Aldo Moro.” Written informed consent to participate in this study was provided by the participants’ legal guardian/next of kin.

## Author contributions

MP: conceptualization, investigation, writing—original draft, and writing—review and editing. GC: conceptualization, methodology, software, formal analysis, investigation, writing—original draft, and writing—review and editing. FD’E: conceptualization, supervision, project administration, and funding acquisition.

## Funding

This work was supported by the European project “STERHEOTYPES—Studying European Racial Hoaxes and Stereotypes” recently founded by “Challenges for Europe” call for Project, Compagnia San Paolo (CUP: B99C20000640007); https://www.irit.fr/sterheotypes/.

## Conflict of interest

The authors declare that the research was conducted in the absence of any commercial or financial relationships that could be construed as a potential conflict of interest.

## Publisher’s note

All claims expressed in this article are solely those of the authors and do not necessarily represent those of their affiliated organizations, or those of the publisher, the editors and the reviewers. Any product that may be evaluated in this article, or claim that may be made by its manufacturer, is not guaranteed or endorsed by the publisher.
