# Supplementary material for: The role of self-efficacy beliefs in dealing with misinformation among adolescents
Source: Front Psychol. 2023 May 18;14:1155280. doi: 10.3389/fpsyg.2023.1155280 (PMC10233930; doi:10.3389/fpsyg.2023.1155280)
Supplement: Supplementary file 1 [file Presentation_1.pdf]

Supplementary Material

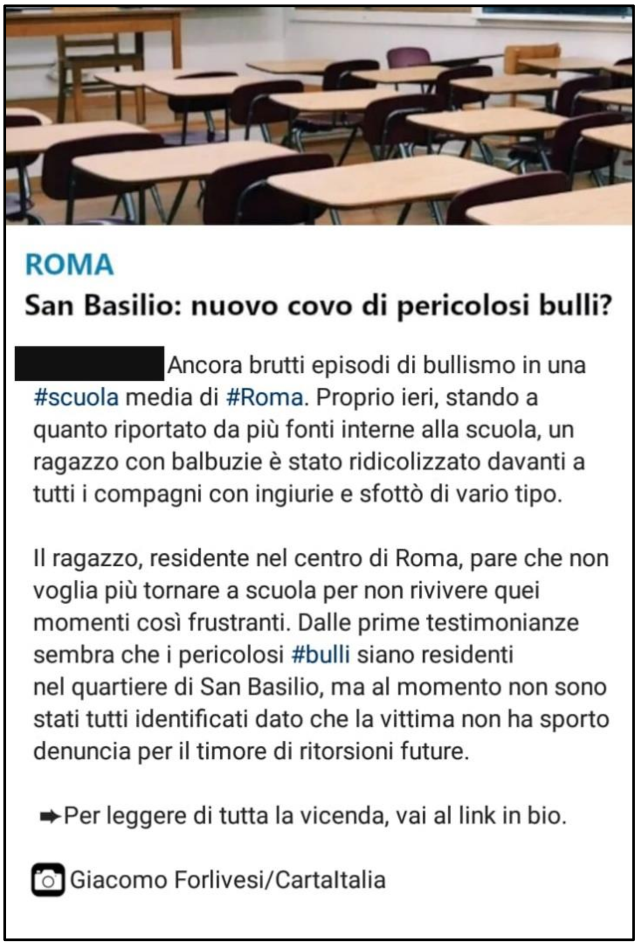

ROMA

San Basilio: nuovo covo di pericolosi bulli?

[redacted] Ancora brutti episodi di bullismo in una #scuola media di #Roma. Proprio ieri, stando a quanto riportato da più fonti interne alla scuola, un ragazzo con balbuzie è stato ridicolizzato davanti a tutti i compagni con ingiurie e sfottò di vario tipo.

Il ragazzo, residente nel centro di Roma, pare che non voglia più tornare a scuola per non rivivere quei momenti così frustranti. Dalle prime testimonianze sembra che i pericolosi #bulli siano residenti nel quartiere di San Basilio, ma al momento non sono stati tutti identificati dato che la vittima non ha sporto denuncia per il timore di ritorsioni future.

➨Per leggere di tutta la vicenda, vai al link in bio.

Giacomo Forlivesi/CartaItalia

Misleading news stimulus example. In the study, the stimulus was framed as if it were a social media screenshot captured with a smartphone. English translation: San Basilio: new hideout of dangerous bullies? More unpleasant incidents of bullying in a #middle school in #Rome. Just yesterday, according to multiple sources inside the school, a boy with a stutter was ridiculed in front of all his classmates with various insults and slurs. The boy, a resident of central Rome, apparently does not want to go back to school to avoid experiencing those frustrating moments again. From the first witness accounts it seems that the dangerous #bullies are residents of the San Basilio neighborhood, but at the moment they have not been identified since the victim has not pressed charges for fear of future retaliation.
